# Radiation exposure per thrombectomy attempt in modern endovascular stroke treatment in the anterior circulation

**DOI:** 10.1007/s00330-020-06837-2

**Published:** 2020-04-24

**Authors:** Charlotte S. Weyland, Fatih Seker, Arne Potreck, Christian Hametner, Peter A. Ringleb, Markus A. Möhlenbruch, Martin Bendszus, Johannes A. R. Pfaff

**Affiliations:** 1 Department of Neuroradiology, Heidelberg University Hospital, Im Neuenheimer Feld 400, 69120 Heidelberg, Germany; 2 Department of Neurology, Heidelberg University Hospital, Im Neuenheimer Feld 400, 69120 Heidelberg, Germany

**Keywords:** Radiation exposure, Fluoroscopy, Thrombectomy, Stroke

## Abstract

**Objective:**

To quantify radiation exposure (RE) of endovascular stroke treatment (EST) in the anterior circulation per thrombectomy attempt and determine causes for interventions associated with high RE.

**Methods:**

A retrospective single-center study of an institutional review board−approved stroke database of patients receiving EST for large vessel occlusions in the anterior circulation between January 2013 and April 2018 to evaluate reference levels (RL) per thrombectomy attempt. ESTs with RE above the RL were analyzed to determine causes for high RE.

**Results:**

Overall, *n* = 544 patients (occlusion location, M1 and M2 segments of the middle cerebral artery 53.5% and 27.2%, carotid artery 17.6%; successful recanalization rate 85.7%) were analyzed. In the overall population, DAP (in Gy cm^2^, median (IQR)) was 113.7 (68.9–181.7) with a median fluoroscopy time of 31 min (IQR, 17–53) and a median of 2 (IQR, 1–4) thrombectomy attempts. RE increased significantly with every thrombectomy attempt (DAP_1_, 68.7 (51.2–106.8); DAP_2_, 106.4 (84.8–115.6); *p* value_1vs2_, < 0.001; DAP_3_, 130.2 (89.1–183.6); *p* value_2vs3_, 0.044; DAP_4_, 169.9 (128.4–224.1); *p* value_3vs4_, 0.001; and DAP_5_, 227.6 (146.3–294.6); *p* value_4vs5_, 0.019). Procedures exceeding the 90th percentile of the attempt-dependent radiation exposure level were associated with procedural complications (*n* = 17/52, 29.8%) or a difficult vascular access (*n* = 8/52, 14%).

**Conclusions:**

Radiation exposure in endovascular stroke treatment is depending on the number of thrombectomy attempts. Radiation exposure doubles when three attempts and triples when five attempts are necessary compared with single-maneuver interventions. Procedural complications and difficult vascular access were associated with a high radiation exposure in this collective.

**Key Points:**

• *Radiation exposure of endovascular stroke treatment (EST) is dependent on the number of thrombectomy attempts.*

• *Reference levels as means for quality control in hospitals performing endovascular stroke treatment should be defined by the number of thrombectomy attempts—we suggest 107 Gy cm*^*2*^*, 156 Gy cm*^*2*^*, 184 Gy cm*^*2*^*, 244 Gy cm*^*2*^*, and 295 Gy cm*^*2*^ *for 1 to 5 maneuvers, respectively, for EST of the anterior circulation*

• *Cases with high rates of radiation exposure are associated with periprocedural complications and difficult anatomical access as a probable cause for a high radiation exposure.*

## Introduction

Radiation exposure by ionizing radiation is a key concern in modern diagnostic radiology with a high physician and patient awareness mainly due to potential relative cancer risk for both parties [1, 2]. As the radiological and neuroradiological CT imaging increased in the last decades, a great effort is made by CT manufacturers, technicians, and physicians to lower radiation exposure [3, 4]. In the 1990s, the term dose reference level (DRL) emerged to control radiation exposure in radiological departments. DRLs showed to effectively control and reduce the radiation exposure for diagnostic imaging [5].

Endovascular stroke treatment (EST) became the standard of care for acute ischemic stroke (AIS) with large vessel occlusion after first-line evidence showed its effectiveness in different prospective multi-center trials in 2015 [6]. Consequently, the number of EST procedures is increasing worldwide [7, 8]. With increasing numbers of neuro-interventional procedures, the interest in tracking and controlling radiation exposure during neuro-interventions is growing [9, 10]. Comparability of procedure quality in differing units, however, may vary. Therefore, following the 7-metric managing approach of the society of vascular and interventional neurology, which includes the control of radiation exposure as the seventh metric, is discussed [11].

The threshold for deterministic risks is reached in about 6% of ESTs while the relevance of the stochastic risk remains uncertain [12]. Although dose area products (DAPs) cannot be directly translated into local skin equivalent doses and should not be mistaken as an equivalent for deterministic risk, DAPs are an established metric to monitor and compare radiation exposure. Monitoring radiation exposure of all stroke centers as a quality control is a practical tool to detect sources of systemically high radiation exposure. Recently, first data emerged to clarify the extent of radiation exposure in EST [12–14]. The number of thrombectomy attempts to recanalize an occluded target vessel is an essential parameter during EST defining the patient’s clinical outcome [15–17].

We hypothesized that radiation exposure increases significantly with the number of thrombectomy attempts during EST. Our objective was to quantify radiation exposure per performed thrombectomy attempt and its relative increase to evaluate, if reference levels should be established per number of thrombectomy attempt in EST. In the second step, we analyzed cases with radiation exposure higher than the 90th percentile in our patient cohort to define reasons for high radiation exposure.

## Methods

In this retrospective single-center study, we report data from an institutional review board–approved stroke database of a university-based comprehensive stroke center. We report data of patients who received EST in the anterior circulation at our comprehensive stroke center consecutively between January 2013 and April 2018. The EST’s radiation exposure was determined (as per dose area product) and subgroups were formed by the number of thrombectomy attempts. A thrombectomy attempt was defined as a planned and conducted maneuver with the intention to recanalize an occluded intracranial vessel.

With the results, reference levels were established by the 75th percentile of each subgroup. In a secondary analysis, cases exceeding the 90th percentile of the radiation exposure per number of thrombectomy attempts were analyzed to define the causes of high radiation exposure during EST.

### Inclusion and exclusion criteria

To achieve comparability between ESTs, patients were selected depending on factors, which directly influenced radiation exposure. Patients were excluded if EST was performed with a monoplane angiographic system or if a complex cervical procedure during the EST was performed (i.e.**,** stent-assisted PTA of the carotid artery). Patient**s** with cervical procedures were excluded due to the diverse approach of EST in a setting of tandem occlusion (e.g.**,** antegrade vs. retrograde approach) leading to a varying predisposal for higher radiation exposure levels. Patients were also excluded, if no EST attempt was performed (e.g.**,** due to futile vascular access). Additionally, only EST procedures performed by an experienced neurointerventionalist (i.e.**,** more than 25 EST procedures performed) were analyzed [8]**.** Since there seems to be no effect of the mode of sedation during EST on fluoroscopy time and radiation exposure, we included patients treated under general anesthesia and conscious sedation [18]**.**

### Performance of EST in the comprehensive stroke center

The decision-making process for EST was achieved in interdisciplinary consensus of the treating neurologist and neurointerventionalist following international and national guidelines. Procedures were performed during and off-hours with a staff consisting of a neurointerventionalist (primary operator), a resident or fellow in interventional radiology (as a scrub assistant), and a medical technical assistant (angio-nurse) to support the procedure.

For EST, a standard approach with femoral access was performed in all cases. In all cases, an 8-F guide catheter and a 5-F or 6-F distal access catheter was used. The choice of material was subject to change depending on the availability and technical progress during the observation period. The choice of material and maneuver technique, i.e., direct aspiration or stent-retriever thrombectomy, was made by the neurointerventionalist and is not further analyzed in this study. The total number of thrombectomy attempts performed during the intervention as specified by the interventionalist in the digitalized interventionalist’s report (mandatory in our department) was verified by reviewing corresponding angiographic images of the procedures.

### Data acquisition

DAP is used to calculate radiation exposure based on the body part irradiated, is a direct indicator of the patient’s effective dose, and already serves as an established paradigm for reference levels. DAP is directly connected to the number of contrast series and road mapping in EST, but it is also influenced by patient positioning, field of view, and individual anatomy. As the relation between DAP and acquisition sequences (contrast runs) is self-evident, it is not further specified in this study. Data acquisition of radiation exposure as per DAP and fluoroscopy time was done automatically by the angiographic systems, which were calibrated regularly. Angiographic systems (Artis Zee Biplane and Artis Q, Siemens Healthineers) underwent technical surveillance with repetitive constancy tests according to German Institute for Standardization (Deutsches Institut für Normung e.V. (DIN)) standards [19, 20]. There was no standardized protocol for additional imaging (e.g., contrast series of the contralateral ICA-territory to delineated collateral flow) beside road mapping and contrast series.

Patient-related data were retrospectively acquired from medical charts and reports and collected in a stroke database approved by the local institutional review board. A separate patient consent was waived for this analysis. Adherence to the STROBE criteria is given [21]. No patient was excluded because of a missing data set.

### Primary outcome parameters

Primary outcome parameters were procedure time, fluoroscopy time in minutes, and radiation exposure per DAP in Gy cm^2^.

### Statistical analysis

Data are shown as median with interquartile range (IQR) or means with standard deviation (SD), as appropriate. After testing for normal distribution using the Shapiro-Wilk test, further analysis was conducted using the Mann-Whitney *U* test, *χ*^2^ test, and one-way non-parametric ANOVA (Kruskal-Wallis test) to compare groups, as appropriate. All tests were performed on the basis of a two-sided level of significance with a *p* value of less than or equal to 0.05 as significant. Statistical analysis was performed by using SPSS Statistics (21.0.0.0; IBM).

## Results

**Fig. 1:**
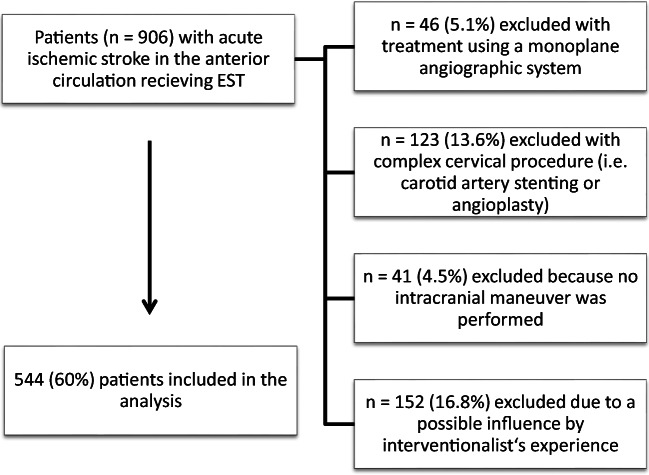
Selection of study cohort (depending on the exclusion criteria)

Of the 906 patients treated with EST between January 2013 and April 2018, *n* = 544 patients met the inclusion criteria for further analysis (see Fig. 1). Thirteen neurointerventionalists were involved in the treatment of the analyzed patients. The median time from stroke onset to groin puncture was 255 min (162–441). Predominantly, occlusions of the main branch of the middle cerebral artery were treated—(M1 occlusions; *n* = 291, 53.5%), followed by occlusions of the M2 segments (*n* = 148, 27.2%) of the MCA. Occlusions of the intracranial carotid-T were less frequent (*n* = 96, 17.6%). Successful reperfusion (mTICI 2b-3) was achieved in *n* = 466 patients (85.7%). For more details, please see Table 1.

**Table 1:** Baseline characteristics of patients who received endovascular stroke treatment entering this analysis

		Patients (*n* = 544)
Age (year), mean (SD)		73 (14)
Male (%)		225 (41.4)
Premorbid Rankin scale mRS^a^		
	0 (%)	200 (36.8)
	1 (%)	118 (21.7)
	2 (%)	101 (18.6)
	3 (%)	93 (17.1)
	4 (%)	19 (3.5)
Initial NIHSS score, median (IQR)		16 (11–20)
Intravenous rtPA (%)		257 (47.2)
Unknown time of symptom onset (%)		176 (32.4)
Time from stroke onset to groin puncture, in minutes, median (IQR)^‡^		255 (162–441)
Procedural aspects		
Type of anesthesia		
Conscious sedation (%)		390 (71.7)
General anesthesia (%)		143 (26.3)
Conversion from conscious sedation to general anesthesia during the procedure (%)		11 (2.0)
Location of intracranial occlusion		
	Carotid T (%)	96 (17.6)
	M1 (%)	291 (53.5)
	M2 (%)	148 (27.2)
	M3 (%)	4 (0.7)
	ACA (%)	5 (0.9)
Number of thrombectomy attempts, median (IQR)		2 (1–4)
Number of thrombectomy attempts, mean (SD)		2.6 (1.9)
Procedure time (groin puncture to last angio-image), in minutes, median (IQR)		61 (38–100)
Dose area product, in Gy cm^2^, median (IQR)		113.7 (68.9–181.7)
Fluoroscopy time, in minutes, median (IQR)		31 (17–53)
Final mTICI score		
	0‑2a (%)	78 (14.3)
	2b (%)	212 (39)
	2c (%)	70 (12.9)
	3 (%)	184 (33.8)

^a^Missing data in 13 patients

^‡^For patients with known symptom onset

*NIHSS*, National Institutes of Health Stroke Scale; *mRS*, modified Rankin Scale score; *mTICI*, modified Thrombolysis In Cerebral Infarction score

### Primary analysis

The median number of thrombectomy attempts was 2 (IQR, 1–4). Overall, the median procedure time was 61 min (38–100) and median fluoroscopy time was 31 min (17–53). Median DAP for all procedures was 113.7 Gy cm^2^ (IQR, 68.9–181.7). DAP was higher than 500 Gy cm^2^ in *n* = 4/544 cases (0.7%); in each case, 5 or more thrombectomy attempts were performed.

Subgroups per number of thrombectomy attempts showed a significant increase in radiation exposure per each additional attempt from 1 to 5 thrombectomy attempts. No difference was observed when comparing EST with five thrombectomy attempts and six or more attempts. Procedures with one or two thrombectomy attempts differ significantly (DAP_1_, 68.7 (51.2–106.8) and DAP_2_, 106.4 (84.8–155.6), *p* value < 0.001). An additional third, fourth, or fifth maneuver led to a significant increase of the radiation exposure as well (DAP_3_, 130.2 (89.1–183.6), *p* value_2vs3_ 0.044; DAP_4_, 169.9 (128.4–244.1), *p* value_3vs4_ 0.001; and DAP_5_, 227.6 (146.3–294.6), *p* value_4vs5_ 0.019; see Table 2).

**Table 2:** Dose area product (DAP) of endovascular stroke treatment according to the number of thrombectomy attempts

Patients receiving mechanical thrombectomy		DAP (Gy cm^2^)				Median increase of DAP due to each additional thrombectomy attempt (Gy cm^2^)	Median increase of DAP due to each additional thrombectomy attempt (%)	*p* value
Number of thrombectomy attempts	Patients, *n*	Mean (SD)	Median	IQR	MIN-MAX			
1	204	88.6 (65.1)	68.7	51.2–106.8	11.6–470.6			
2	122	123.8 (60.9)	106.4	84.8–155.6	31.2–456.9	37.7	54.9	< 0.001
3	79	143.4 (71.8)	130.2	89.1–183.6	41–391.5	23.9	22.4	0.044
4	59	181.8 (75.5)	169.9	128.4–244.1	60.1–375.3	39.7	30.5	0.001
5	31	246.3 (140.7)	227.6	146.3–294.6	73.7–773.6	57.6	33.9	0.019
≥ 6	49	261.6 (115.9)	242.2	183.8–332.7	62–597	14.7	6.4	0.346

Likewise, fluoroscopy time increased per each additional thrombectomy attempt and was significantly different when comparing EST with one or two thrombectomy attempts (median (IQR), in minutes; FT_1_, 16 (11–29) and FT_2_, 26 (21–41), *p* value 0.001). An additional third, fourth, or fifth thrombectomy attempt led to a significant increase (FT_3_, 35 min (25–45), *p* value_2vs3_ 0.008; FT_4_, 49 min (32–76), *p* value_3vs4_ 0.0003; and FT_5_, 67 min (57–98), *p* value_4vs5_ 0.005). There was no difference when comparing EST with five thrombectomy attempts or six and more (see Table 3).

**Table 3:** Fluoroscopy time (FT) of endovascular stroke treatment according to the number of thrombectomy attempts

Patients receiving mechanical thrombectomy		FT (min)				Median increase of FT due to each additional thrombectomy attempt (min)	Median increase of FT due to each additional hrombectomy attempt (%)	*p* value
Number of thrombectomy attempts	Patients, *n*	Mean (SD)	Median	IQR	MIN-MAX			
1	204	23 (22)	16	11–29	4–131			
2	122	34 (22)	26	21–41	4–117	10	62.5	< 0.001
3	79	40 (24)	35	25–45	9–143	9	34.6	0.008
4	59	55 (30)	49	32–76	18–138	14	40	0.001
5	31	84 (55)	67	57–98	20–304	18	36.7	0.002
≥ 6	49	80 (34)	70	55–98	36–176	3	4.5	0.988

Based on our results, radiation exposure values of the 75th percentile were set as a reference level per number of thrombectomy attempts for this patient cohort and the 90th percentile of this patient cohort was defined likewise (see Table 4).

**Table 4:** 75th percentile (reference levels) and 90th percentile of the dose area product in Gy cm^2^ per number of thrombectomy attempts in this cohort

	Thrombectomy attempts					
	1	2	3	4	5	> 5
P_75_	107	156	184	244	295	333
P_90_	145	196	227	273	407	419

### Secondary analysis

Analyzing patients with a thrombectomy-attempt-dependent radiation exposure above the 90th percentile (*n* = 52/544 patients, 9.6%), a difficult anatomical access and periprocedural complications (vessel dissection/perforation or vasospasms) were found as main factors prolonging the procedure, requiring additional imaging and thereby increasing radiation exposure (see Table 5). Periprocedural complications (*n* = 18/52, 34.6%) included vasospasms, vessel dissection, or perforation needing further imaging for surveillance and occasionally treatment. Difficult anatomical access comprises extracranial and intracranial vessel tortuosity and variants leading to a longer time to reach the occluded vessel in *n* = 11/52 (21.2%) patients. In *n* = 8/52 cases (15.4%), additional imaging was needed for diagnostic series contralaterally and in 15/52 cases (28.8%), a reason for high radiation exposure could not be identified retrospectively.

**Table 5:** Reasons for endovascular stroke treatment procedures exceeding the 90th percentile of radiation exposure in this single-center analysis (*n* = 52)

	Number of thrombectomy attempts						
	1	2	3	4	5	> 5	*n* (total)
Number	20	12	8	5	2	5	52
Reasons							
Vasospasms	4	1	0	0	0	1	6
Vessel dissection/perforation	4	4	3	1	0	0	12
Anatomically difficult access	2	3	2	2	2	0	11
Additional imaging (e.g., contralateral ICA-territory)	4	1	1	0	0	2	8
Unknown	6	3	2	2	0	2	15

*ICA*, internal carotid artery

## Discussion

With this analysis, we report radiation exposure during EST in the anterior circulation and derive reference levels depending on the number of thrombectomy attempts for 1 to 5 EST-attempts from the so far largest single-center patient cohort (*n* = 544). As expected, a stepwise increasing radiation exposure and fluoroscopy time from thrombectomy attempts 1 to 5 were observed. The extent of this increase in radiation exposure is surprising.

In a previous single-center study of *n* = 319 patients, Farah et al analyzed radiation exposure of EST with the objective of identifying factors influencing radiation exposure [13]. They report an overall median DAP for EST of 94 Gy cm^2^ with a median number of thrombectomy attempts of 2 and suggests a reference level for all procedures of 162 Gy cm^2^ [13]. These data are comparable with the overall median DAP for EST observed in the patient cohort reported in the current manuscript. Farah and his coauthors suggest that the number of thrombectomy attempts is a factor influencing radiation exposure, but they do not propose reference levels by thrombectomy attempts. The 75th percentile of DAP was merely given for 1 and 2 attempts and reported to be 100 Gy cm^2^ and 158 Gy cm^2^, which is comparable with our data. However, there are methodical differences as in the publication by Farah et al: (i) ESTs in both the anterior and posterior circulation were included, (ii) complex cervical procedures were not excluded or reported separately, and (iii) the possible influence by the interventionalist’s experience [8] was not reported separately.

Another comparable, multi-center study by Guenego et al focused on the reduction of radiation exposure by using a specific radiation dose-reduction software in *n* = 520 procedures compared with *n* = 576 ESTs without such a system. In this previous study, reference levels were determined for ESTs with the dose-reduction software in use as 148 Gy cm^2^ and without using the software as 187 Gy cm^2^. There is no specification regarding the radiation exposure per number of thrombectomy attempts [14]. The reference level for interventions without a dose-reduction system is therefore as high as the reference level for ESTs with 3 thrombectomy attempts in the current study. According to our data, radiation exposure of ESTs with more than three thrombectomy attempts (performed in *n* = 139/544 (25.6%) patients) is on average higher than the reference level proposed by Guenego et al.

In our analysis, radiation exposure nearly doubles when three thrombectomy attempts are necessary compared with single-maneuver interventions. Our results also show a comparably high effect when four or five thrombectomy attempts are necessary instead of three. Establishing reference levels depending on the number of thrombectomy attempts appears reasonable, as an EST with four thrombectomy attempts, i.e., every tenth procedures in this study, showed a median DAP of 170 Gy cm^2^ and thereby exceeded the overall reference level previously proposed by Farah et al. We did not differentiate the thrombectomy attempts further, because ESTs with more than 5 attempts were rear in our patient cohort and in accordance with the original idea that reference levels at least 25 cases per subgroup should be evaluated in order to establish reference levels. The reference levels provided in this analysis only serve as thresholds for this single-center experience. To establish generally valid reference levels for EST, a multi-center study is inevitable.

When reference levels per number of thrombectomy attempts are used in the clinical context, stroke centers can search for reasons of high radiation exposure. Above the 90th percentile of this patient cohort, we identified procedural complications, including vessel dissection or perforation and vasospasms as well as an anatomical difficult vascular access as possible reasons for high radiation exposure. In *n* = 15/52 (28.8%) cases with a radiation exposure above the 90th percentile, a probable cause could not be found retrospectively. A direct feedback for the neurointerventionalist after the intervention if the applied radiation exposure lies above a certain level can lead to better awareness and a better understanding of the reasons causing high radiation exposure. Documentation of causes of high radiation exposure by the neurointerventionalist after the procedure would also serve as a training of the neurointerventionalist and contribute to quality assurance. Procedural complications and anatomically difficult vessel accesses are probably also among the patients below the 90th percentile of radiation exposure but were not further evaluated in this study. If present, these complications and access difficulties did not lead to a high radiation exposure; e.g., mild vasospasms are often self-limiting and do not always require treatment or more imaging surveillance during EST.

The authors of this article want to point out that EST should not be withheld from eligible patients or stopped because a certain radiation exposure is reached. Reference levels do not represent limits to stop a treatment. Foremost and under consideration of the patients’ safety, it remains important to recanalize intracranial vessel occlusions in order to give patients the best chance for clinical recovery according to international guidelines [22]. While further research seems necessary to characterize radiation exposure during EST in different settings, extraordinary complicated or challenging cases of EST will be related to a higher radiation exposure, unless imaging technique during the intervention itself can be changed.

### Limitations

Limitations of this study are mainly related to its single-center retrospective design. Therefore, generalizability of our results is limited and depending on the local setting. While we can show that radiation exposure during EST is dependent on the number of thrombectomy attempts, which appears to be plausible to apply to other settings as well, reference levels for EST should be further investigated with a multi-center approach. The use of a dose-reduction system was not given in this study but can decrease radiation exposure and thereby also contribute to the control of radiation exposure in EST in comprehensive stroke centers [14].

We also did not obtain the patients’ body mass index as an influencing factor of radiation exposure, which is influencing the radiation exposure mainly in diagnostic and interventional imaging of the thorax and abdomen, but has a minor influence in head and neck procedures [23]. On the other hand, Miller et al suggested to establish reference levels in interventional neuroradiology without a weight correction [10].

## Conclusion

This single-center retrospective study of *n* = 544 patients reports a significantly increased radiation exposure during EST in the anterior circulation depending on the number of thrombectomy attempts. For ESTs with one to five thrombectomy attempts, establishing reference levels per thrombectomy attempt is feasible.

Procedural complications and difficult anatomical vessel access are deciphered as possible causes for a high radiation exposure in this cohort. Establishing reference levels and further investigating for reasons of higher radiation exposure can be useful as general operating standard in angiography suites and thereby become a quality control standard in comprehensive stroke centers.

## Abbreviations

AIS: Acute ischemic stroke
DAP: Dose area product
DIN: Deutsches Institut für Normung
DRL: Dose reference level
EST: Endovascular Stroke Treatment
FT: Fluoroscopy time
IQR: Interquartile range
mRS: Modified Rankin scale
mTICI: Modified thrombolysis in cerebral infarction score
RE: Radiation exposure
RL: Reference level
